# Measuring, valuing, and incorporating patient and caregiver productivity costs in economic evaluations: a scoping review and environmental scan

**DOI:** 10.1017/S0266462326103882

**Published:** 2026-06-26

**Authors:** Shant Torkom Yeretzian, Carly Sillcox, Hannah Loshak, Lauren Ramsay, Alex Gregory Rodriguez Barizonte, Emma Shapiro, Yeva Sahakyan, Beate Sander

**Affiliations:** 1 Institute of Health Policy, Management and Evaluation, University of Toronto, Canada; 2 Health Systems and Policy Research Collaborative Centre, University Health Network, Canada; 3 School of Medicine, Trinity College Dublin, Ireland; 4 Canada’s Drug Agency, Canada; 5 Faculty of Arts and Science, University of Toronto, Canada; 6 Faculty of Health Sciences, Queen’s University, Canada; 7 ICES, Canada

**Keywords:** productivity, measurement, valuation, presenteeism, absenteeism

## Abstract

**Objectives:**

Economic evaluations adopting societal perspectives often include productivity costs, yet these remain inconsistently measured and valued. We aimed to identify existing measurement instruments, valuation methods, and guidance for incorporating productivity costs in economic evaluations.

**Methods:**

We conducted a scoping review of peer-reviewed literature (MEDLINE, Embase, EconLit; 2019–2025) to map methods for measuring and valuing productivity costs, and an environmental scan of grey literature to identify complementary policy and methodological guidance from Health Technology Assessment organizations. Two reviewers independently screened records and extracted data on study characteristics, instruments, valuation methods, and recommendations. We synthesized findings descriptively and thematically.

**Results:**

We screened 3,971 records and included 40 studies: 29 on measurement instruments and 11 on valuation methods. Among twenty-five instruments, twenty-three measured productivity loss for patients, two for caregivers; thirteen measured presenteeism only, four captured presenteeism and absenteeism, and eight measured presenteeism, absenteeism, and unpaid work; three were suitable for valuation using the human capital approach (HCA) and friction cost approach (FCA). Studies highlighted limitations, including HCA’s tendency to overestimate and FCA’s reliance on scarce friction period data. We also identified ten guidance documents: several jurisdictions favored FCA to value paid labor (*n* = 4) and opportunity cost for unpaid labor (*n* = 3).

**Conclusions:**

There are many productivity cost instruments, but only few are suitable for valuation. Valuation remains contested, and guidance is fragmented: HCA is simple but risks overestimation, whereas FCA better reflects labor markets but requires data on friction periods. Future work should prioritize valuation-ready tools, improved friction period estimation, and harmonized guidance.

## Highlights

- This study synthesizes methods for measuring, valuing, and incorporating patient and caregiver productivity costs by integrating evidence from peer-reviewed literature and international Health Technology Assessment guidance.
- We identify twenty-five instruments, but only three capture the information required for valuation using the human capital or friction cost approach and summarize current recommendations for measuring and valuing paid and unpaid labor across jurisdictions.
- Our findings highlight the need for valuation-ready instruments, better friction period estimation, and more harmonized guidance to improve the comparability and policy relevance of societal-perspective economic evaluations.

## Introduction

Economic evaluations play a central role in informing healthcare decision-making by comparing the costs and consequences of alternative interventions (1). Beyond health outcomes and direct medical expenditures, illness and treatment can disrupt individuals’ ability to work, reduce productivity while at work, and create demand for unpaid caregiving, all of which carry economic consequences not captured by health outcomes alone (2;3). These impacts carry economic consequences that may fall outside the healthcare system (4). Recognizing the importance of capturing consequences beyond health, the Second Panel on Cost-Effectiveness in Health and Medicine recommended that economic evaluations adopt a societal perspective in the reference case alongside the health sector perspective (5). The societal perspective captures health system costs and costs that fall outside the health sector, such as those borne by patients and families, as well as productivity losses from paid and unpaid work (6). A key implication of this recommendation is that productivity costs – losses in paid and unpaid work due to illness, disability, treatment, and premature death – should be incorporated directly into the numerator of the cost-effectiveness ratio, that is, as part of the incremental costs considered in analysis (5).

Productivity costs encompass losses in paid and unpaid work (7). For paid work, they arise through presenteeism and absenteeism, which refer to reduced productivity at work and absence from work, respectively (7). For unpaid work, they involve time devoted to informal caregiving or household production that is displaced by illness or treatment (8). Productivity costs are calculated through the measurement and valuation of productivity losses. Measurement quantifies time lost due to presenteeism and absteeism, using self-report instruments (e.g., Work Productivity and Activity Impairment Questionnaire (WPAI)). Valuation assigns a monetary value to this lost time. For paid work, it is commonly conducted through the human capital approach (HCA), which values all lost time over the full duration of absence at the individual’s wage rate, assuming no replacement of the worker, or the friction cost approach (FCA), which limits costs to the period required to replace a worker (friction period), reflecting labor market dynamics such as replacement of workers and the availability of unemployed labor (9–12). For unpaid work, valuation methods include the replacement cost approach, which estimates the expense of hiring someone to perform the same tasks, or the opportunity cost approach, which values time based on forgone paid work (11).

Despite their recognized importance, considerable variability exists in how productivity costs are measured and valued across economic evaluations (9). Variation exists in which types of losses are included, the instruments used to capture time losses, and the methods applied to assign monetary values (11). These differences can substantially affect estimates of cost-effectiveness and limit comparability across studies and jurisdictions. Previous reviews have examined aspects of productivity cost measurement and valuation. Hubens et al. systematically reviewed productivity loss measurement instruments and their suitability for economic evaluation, while Zhang et al. (12) and Mamiya et al. examined how productivity outcomes are measured and incorporated in randomized controlled trials and economic evaluations (9;12;14). Building on this work, we aimed to conduct a scoping review of peer-reviewed literature and an environmental scan of guidance documents (e.g., Health Technology Assessment (HTA) reports, national guidelines, white papers, and policy documents) and synthesize existing methods, recommendations, and consensus for measuring, valuing, and incorporating patient and caregiver productivity costs in economic evaluations.

## Methods

### Study design

We conducted a two-part study comprising a scoping review and an environmental scan to identify and synthesize existing methods and guidance on the measurement and valuation of patient and caregiver productivity costs in the context of economic evaluations. The scoping review focused on peer-reviewed literature and followed a structured framework to map and describe how productivity costs are measured and valued in health economic research. The environmental scan was conducted in parallel to identify guidance on measurement, valuation, and incorporation of productivity costs in economic evaluations from grey literature sources, including policy documents, HTA guidelines, and methodological reports from Canadian and international organizations. Below, we detail the methods for each component.

### Scoping review

We followed the six-stage methodological framework developed by Arksey and O’Malley and updated by Levac et al. and the JBI (15;16). The scoping review protocol has been registered on Open Science Framework (https://doi.org/10.17605/OSF.IO/FK9D4) and published in BMJ Open (17).

#### Research questions

The review was guided by the overarching research question: *How should patient and caregiver productivity be measured, valued, and incorporated into economic evaluations?* To address this, we sought literature providing methodological contributions in two main areas: methods for measuring patient and caregiver productivity losses, including changes in paid and unpaid labor due to morbidity, disability, treatment, or mortality and methods for valuing lost productivity, including the HCA and FCA, along with recommendations regarding their application.

#### Search strategy

The information specialist developed the search strategy using the Population–Concept–Context framework (18), where the population was patients and caregivers, the concept was productivity costs, and the context was literature on their measurement and valuation. The search targeted peer-reviewed studies on methods for assessing productivity loss (absenteeism, presenteeism, and unpaid labor) and approaches for valuation. Searches were conducted in MEDLINE, Embase, and EconLit for publications from January 1, 1996 through July 9, 2025 (19). The 1996 start date corresponds to the release of the US Public Health Service Panel’s cost-effectiveness guidelines, which established the reference-case framework that continues to shape current economic evaluation methods (19). See Supplementary Appendix 2 for search strategies by database.

#### Eligibility criteria

**Figure 1.**
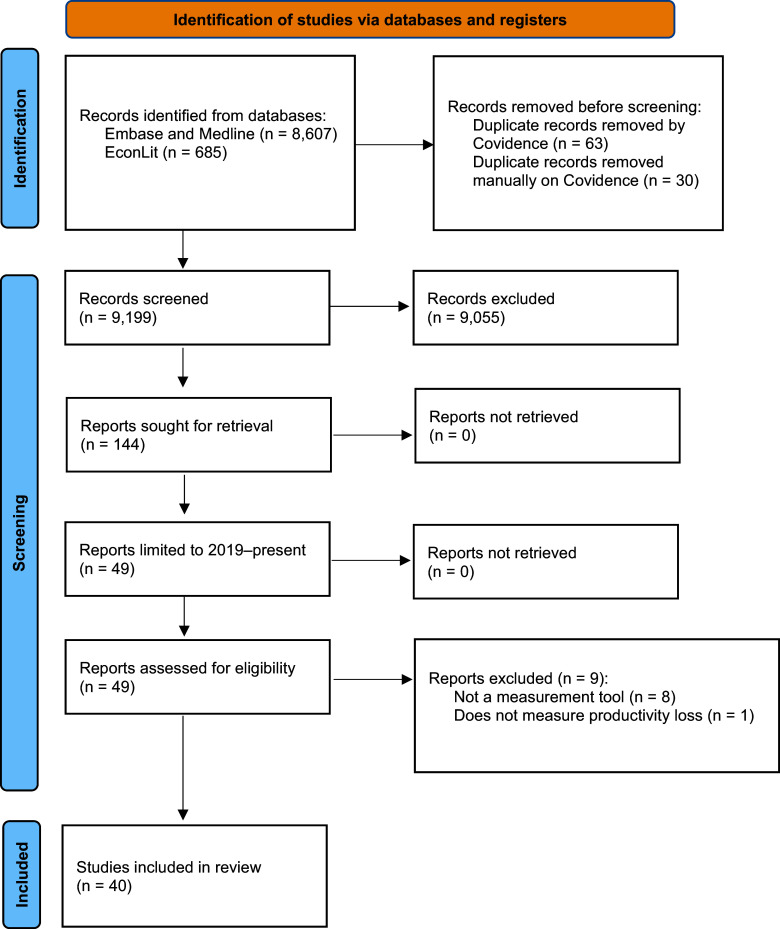
Preferred reporting items for systematic reviews and extended meta-analyses (PRISMA) 2020 flow diagram. Figure 1. long description.

After deduplication, using Ovid deduplication for multifile searches and Endnote, and import to Covidence, pairs of reviewers (CS, AGRB, ES, STY) independently screened titles, abstracts, and full texts against predefined criteria (20;21). We included studies that contributed to methods for measuring or valuing productivity costs, such as the development, adaptation, or validation of measurement instruments, or comparisons of valuation approaches. We excluded studies limited to estimating productivity losses in particular disease settings without methodological contribution. During the full-text screening stage, we limited inclusion to reports published from 2019 onward after identifying a comprehensive review of earlier literature by Hubens et al. (9). We excluded applied economic evaluations, as they have been reviewed elsewhere (9;14). However, for completeness, we extracted and summarized instruments identified in those reviews. Disagreements during screening were resolved by discussion amongst the reviewers, with arbitration by a third reviewer (STY, BS) when needed. Reasons for exclusion of full-texts were recorded and presented in the PRISMA flow diagram (see Figure 1).

#### Data extraction and summary

We used a standardized data extraction form in Covidence to extract information on bibliographic details (author name, year of publication, country, and study design), study design, and methodological focus (21). For studies on measurement instruments, we extracted information on the measurement instrument (e.g., WPAI, Work Limitation Questionnaire), population (patients or caregivers), assessment of validity and reliability, type of productivity loss (e.g., absenteeism, presenteeism, unpaid labor), the instrument’s ability to quantify productivity loss in time-based units (e.g., hours or days), and suitability for monetary valuation using the HCA or FCA. Instruments were considered suitable for the HCA if they provided information on long-term disability or extended work absence, and for the FCA if they provided information on the frequency and duration of absence episodes, to account for the initiation of friction periods (9). For studies reporting on valuation methods, we extracted information on methods and any recommendations provided regarding their use. The data extraction form was pilot tested and updated iteratively as new concepts emerged. Pairs of reviewers (CS, AGRB, ES, STY) independently extracted data from all included studies, with discrepancies resolved through discussion or arbitration by a third reviewer (STY, BS).

We applied a descriptive analytical approach to summarize and report the findings. We synthesized the extracted data narratively and organized them thematically. We presented measurement instruments and valuation methods separately, highlighted patterns and similarities, and noted areas of divergence to map the current landscape and inform future practice. We reported the results in line with the PRISMA-ScR guidelines (Supplementary Appendix 1) (22).

#### Consultation

We developed the research question and methodological approach in collaboration with partners from Canada’s Drug Agency (CDA-AMC), with expertise in health economics, to ensure alignment with the needs of HTA decision-making and current policy considerations related to the inclusion of productivity costs in economic evaluations. We then conducted a focused consultation with our CDA-AMC partners to assess the policy relevance of our findings. The consultation involved online meetings and review of the manuscript, with iterative feedback on the study design, preliminary results, and interpretation. Discussions focused on clarity, feasibility, and applicability of measurement and valuation methods in evaluations conducted from a societal perspective. We incorporated the feedback to refine the analysis and presentation of findings.

### Environmental scan

To complement the scoping review and identify consensus-based guidance, methodological recommendations, and relevance to policy and HTA, we conducted a targeted environmental scan of grey literature sources focused on the measurement, valuation, and incorporation of productivity costs into economic evaluations. We aimed to capture relevant policy documents, technical reports, HTA guidelines, and other nonindexed materials published by HTA bodies, government agencies, international organizations, and academic institutions.

We systematically searched the websites of major national and international HTA organizations known for producing health economic and HTA guidance, such as CDA-AMC and Institute for Clinical and Economic Review (ICER). See Supplementary Appendix 3 for the full list of sources searched. We conducted Google searches (date: July 17, 2025) using combinations of terms such as productivity loss, productivity costs, absenteeism, presenteeism, unpaid work, economic evaluation, HTA, grey literature, and methodological guidance. The first 5 pages of results from each query were screened to identify potentially relevant documents.

Pairs of reviewers (CS, AGRB, ES, STY) independently screened the identified documents for eligibility. Documents were included if they discussed or provided guidance on methods for measuring or valuing productivity costs in the context of health economic evaluations. We excluded documents that focused solely on labor market productivity, workplace efficiency, or unrelated economic impacts outside the health context. Screened documents were reviewed in full text, and relevant content was extracted using the same data extraction form developed for the peer-reviewed literature, with minor adaptations to reflect the structure of policy and guidance documents. Recommendations and rationales were recorded verbatim when available and categorized thematically.

### Ethics approval

This review relied exclusively on publicly available literature and did not involve human participants or the use of primary data; therefore, ethics approval was not required.

## Results

### Study characteristics

The peer-reviewed literature search yielded 9,292 records. After removing 93 duplicates, 9,199 titles and abstracts were screened, of which 9,055 were excluded. After limiting the records to 2019–present, 49 full-text articles were assessed for eligibility, of which 40 were included in the review. The PRISMA flow diagram is presented in Figure 1. Twenty-six of the included studies reported twenty-five measurement instruments, primarily reporting the development (*n* = 8), validation (*n* = 12), or adaptation (*n* = 8) of instruments for measuring productivity loss. Eleven studies explored valuation methods. Seven studies were reviewed: two were systematic reviews of economic evaluations, two were systematic reviews reporting valuation methods, one was a scoping review of randomized controlled trials measuring and reporting productivity losses, and two were reviews reporting valuation methods. Most studies were conducted in Europe (*n* = 16), Asia (*n* = 14), and North America (*n* = 6). The study characteristics are detailed in Table 1.

**Table 1.** Characteristics of included studies Table 1. long description.

Author, year	Country	Study design	Measurement instruments and valuation methods
**Studies exploring measurement instruments**			
Abdi et al., 2021 (50)	Iran	Instrument development	Persian Stanford Presenteeism Scale (P-SPS-6)
Abma et al., 2019 (51)	Netherlands	Instrument development and validation	Work Role Functioning Questionnaire (WRFQ) short forms: WRFQ 5, WRFQ 10
Ademiluyi et al., 2025 (52)	Netherlands	Instrument development	Institute for Medical Technology Assessment (iMTA) productivity cost questionnaire (iPCQ) and short forms
Avance et al., 2024 (53)	Brazil	Instrument validation	Work Limitation Questionnaire (WLQ)
Baldonedo-Mosteiro et al., 2020 (54)	Spain	Instrument validation	Stanford Presenteeism Scale-6 (SPS-6)
Baris et al., 2023 (55)	Turkey	Instrument development and validation	Sickness Presenteeism Scale-Nurse (SPS-N)
Beemster et al., 2019 (26)	Netherlands	Instrument validation	Institute for Medical Technology Assessment (iMTA) productivity cost questionnaire-vocational rehabilitation (iPCQ-VR)
Bezzina et al., 2023 (56)	Poland	Instrument validation	Six-item Stanford Presenteeism Scale (SPS-6)
D’Amico et al., 2020 (57)	Italy	Instrument development/validation	The Migraine Work and Productivity Loss Questionnaire (MWPLQ), the Work Productivity and Activity Impairment (WPAI), the Headache Impact Test (HIT-6), the Nigraine Disability Assessment Score (MIDAS), HEADWORK Questionnaire
Ford et al., 2023 (58)	United States	Instrument validation	Work Productivity and Activity Impairment (WPAI) Specific Health Problem version 2 questionnaire, Migraine Specific Quality of Life Questionnaire version 2.1 (MSQv2.1)
Gelfand et al., 2021 (8)	Canada	Instrument adaptation	Valuation of Lost Productivity (VOLP) questionnaire, Work Productivity and Activity Impairment (WPAI) questionnaire
Hubens et al., 2021 (9)	Netherlands	Systematic review	Forty-two instruments
Ishimaru et al., 2020 (59)	Japan	Instrument validation	Work Functioning Impairment Scale (WFun)
Jiang et al., 2020 (47)	China	Instrument adaptation	Stanford Presenteeism Scale (SPS-6) (Chinese version)
Kim et al., 2020 (60)	South Korea	Instrument adaptation	Institute for Medical Technology Assessment Productivity Cost Questionnaire (iPCQ) (Korean version) Work Productivity and Activity Impairment Questionnaire (WPAI) (Korean version)
Kawakami et al., 2020 (61)	Japan	Instrument adaptation	World Health Organization Health and Performance Questionnaire (WHO-HPQ) (Japanese version)
Landfeldt et al., 2019 (46)	Sweden	Instrument development	The Caregiver Indirect and Informal Care Cost Assessment Questionnaire (CIIQ)
Lui and Johnston, 2019 (62)	Hong Kong	Instrument development	Multidimensional Presenteeism Exposures and Productivity Survey for Nurses (MPEPS-N) (Chinese version)
Mamiya et al., 2025 (14)	Japan	Systematic review	Twenty instruments
Pokrzywinski et al., 2020 (63)	United States	Instrument validation	Health-Related Productivity Questionnaire, version 2 (HRPQ)
Seo et al., 2024 (64)	South Korea	Instrument adaptation	Stanford Presenteeism Scale-6 (shortened by two items) (Korean version) (K-SPS-4)
Seyedrezazadeh et al., 2020 (65)	Iran	Instrument adaptation	Work Productivity and Activity Impairment Questionnaire (Persian version) (WPAI-AQ)
Ta-Thi and Chuang 2022 (66)	Taiwan	Instrument validation	Work Productivity and Activity Impairment Questionnaire (Chinese version) (C-WPAI) General Health and the World Health Organization’s Health and Work Performance Questionnaire (Chinese version) (C-WHO-HPQ)
Ta-Thi and Chuang, 2022 (67)	Taiwan	Instrument adaptation	Stanford Presenteeism Scale-6 (Chinese version) (CSPS-6)
Teoman and Harmanci Seren, 2022 (68)	Turkey	Instrument adaptation	Stanford Presenteeism Scale-Short Form (Turkish version)
Van der Burg et al., 2020 (69)	Netherlands	Instrument validation	Work Ability Index (WAI), Quantity and Quality (QQ) method, Osterhaus-method
Wu et al., 2019 (70)	United States	Methodological examination	Work Productivity Impairments Questionnaire (for Psoriasis) (WPAI-PsO)
Zhang et al., 2023 (71)	Canada	Scoping review	Productivity and Disease Questionnaire (PRODISQ), Quantity and Quality method (QQ), Short Form Health and Labor Questionnaire (SF-HLQ), Treatment Inventory of Costs in Patients with Psychiatric Disorders (TiC-P), Work Ability Index (WAI), World Health Organization Health and Work Performance Questionnaire (WHO-HPQ), Work Limitations Questionnaire (WLQ), Work Productivity and Activity Impairment Questionnaire (WPAI), Work Productivity Survey (WPS)
Zhu et al., 2019 (72)	Canada	Instrument development	Work hoarse
**Studies exploring valuation methods**			
Brouwer et al., 2023 (7)	Netherlands	Case study	Multiplier effects, compensation mechanisms
Grega and Kolář, 2021 (31)	Czech Republic	Review	HCA, FCA
Hanly et al., 2019 (29)	Ireland	Case study	HCA, FCA
Hanly et al., 2020 (33)	Ireland	Case study	FCA
Hanly et al., 2023 (32)	Ireland	Case study	FCA
Jiang et al., 2022 (73)	China	Review	FCA, HCA
Krol et al., 2023 (13)	Netherlands	Systematic review	Multiplier effects, compensation mechanisms
Manipis et al., 2021 (34)	Australia	Case study	FCA
Manipis et al., 2025 (30)	Australia	Case study	FCA, HCA
Rissanen et al., 2021 (35)	Finland	Case study	HCA, FCA
Setiawan et al., 2024 (28)	Australia	Systematic review	HCA, FCA

Abbreviations: FCA, friction cost approach; HCA, human capital approach; QALY, quality-adjusted life years.

### Measurement instruments

We identified twenty-five unique instruments for measuring productivity loss across forty studies (Table 2). Eighteen were general-use, and seven were disease-specific; most were designed for patients (*n* = 23), while two were developed for caregivers. All were self-administered, with recall periods ranging from 7 days to 3 months, with a 4-week recall period being the most common (*n* = 11). Nineteen instruments demonstrated validity, and fourteen demonstrated reliability. All were available in English, and thirteen had translations into three or more languages.

**Table 2.** Productivity loss measurement instruments Table 2. long description.

Instrument name	Generic or disease-specific	Mode of administration	Languages available	Population	Validity/ reliability	Productivity cost components captured	Recall period	Does it allow for quantification?	Valuation suitability	Reference(s)
**Instruments developed for caregivers**										
The Caregiver Indirect and Informal Care Cost Assessment Questionnaire (CIIQ)	Generic	Self-reported	English, German, French, Italian, Danish, Norwegian, Finnish, Swedish	Caregiver	NA/NA	Absenteeism, presenteeism, unpaid work	1 week	Yes, for absenteeism and presenteeism	No	Landfeldt et al., 2019 (46)
Valuation of Lost Productivity (VOLP) Questionnaire	Generic	Self-reported	English, Dutch, French, German, Italian, Spanish, Arabic, Romanian, Polish, Hungarian, Norwegian, and Russian	Caregiver	Yes/NA	Absenteeism, presenteeism, unpaid work	3 months (absenteeism) and also 7 days (presenteeism)	Yes, for absenteeism, presenteeism, and unpaid work	No	Gelfand et al., 2021 (8)
**Instruments developed for patients**										
Headache Impact Test (HIT-6)	Disease-specific	Self-reported	English, Italian	Patient	NA/NA	Presenteeism	4 weeks	Yes, for presenteeism	No	D’Amico et al., 2020 (57)
HEADWORK questionnaire	Disease-specific	Self-reported	Italian, English, Dutch, Portuguese, German	Patient	Yes/Yes	Presenteeism	4 weeks	Yes, for presenteeism	No	D’Amico et al., 2020 (57)
Health-Related Productivity Questionnaire, version 2 (HRPQ)	Generic	Self-reported	English	Patient	Yes/NA	Absenteeism, presenteeism, unpaid work	1 week	Yes, for absenteeism, presenteeism, and unpaid work	No	Pokrzywinski et al., 2020 (63)
iMTA Productivity Cost Questionnaire (iPCQ)	Generic	Self-reported	Arabic, English, Dutch, Czech, Danish, Finnish, French, German, Hungarian, Italian, Korean, Norwegian, Polish, Portuguese, Romanian, Spanish, Turkish	Patient	Yes/Yes	Absenteeism, presenteeism, unpaid work	4 weeks	Yes, for absenteeism, presenteeism, and unpaid work	Supports HCA; Supports FCA	Ademiluyi et al., 2025 (52); Beemster et al., 2019 (26); Mamiya et al., 2025 (14); Kim et al., 2020 (60)
iMTA Productivity Cost Questionnaire-Vocational Rehabilitation (iPCQ-VR)	Generic	Self-reported	English, French, Dutch	Patient	Yes/Yes	Absenteeism, presenteeism, unpaid work	4 weeks	Yes, for absenteeism, presenteeism, and unpaid work	Supports HCA; Supports FCA	Beemster et al., 2019 (26)
Migraine Work and Productivity Loss Questionnaire (MWPLQ)	Disease-specific	Self-reported	English, Italian	Patient	NA/NA	Presenteeism, absenteeism	Duration of the respondent’s most recent migraine headache	Yes, for absenteeism, presenteeism, and unpaid work	No	D’Amico et al., 2020 (57)
Multidimensional presenteeism exposures and productivity survey for nurses (MPEPS-N) (Chinese version)	Generic	Self-reported	English, Cantonese	Patient	Yes/Yes	Presenteeism	4 weeks	Yes, for presenteeism	No	Lui and Johnston, 2019 (62)
The Migraine Disability Assessment Score (MIDAS)	Disease-specific	Self-reported	English	Patient	NA/NA	Presenteeism, absenteeism	3 months	Yes, for absenteeism, presenteeism, and unpaid work	No	D’Amico et al., 2020 (57)
Osterhaus-method	Generic	Self-reported	English, Dutch, French, German, Italian, Spanish, Portuguese, Japanese, Chinese (Simplified), Korean, Russian, Turkish, Polish, Arabic, Hindi, Thai, Vietnamese, Indonesian, Czech, Greek, Hungarian, Romanian, Swedish, Norwegian, Finnish, Danish, Bulgarian, Serbian, Croatian, Lithuanian, Estonian, Latvian, Slovenian, Slovak, Ukrainian, Hebrew, Malay, Filipino, Bengali, Tamil, Telugu, Kannada, Gujarati, Punjabi, Marathi, Urdu, Nepali, Sinhala, Khmer, Lao, Burmese, Mongolian, Fijian, Samoan, Tongan, Hawaiian, Maori, Tahitian	Patient	Yes/NA	Presenteeism	2 weeks	Yes, for presenteeism	No	Van der Burg et al., 2020 (69)
Quantity and Quality (QQ) method	Generic	Self-reported	English, German, French, Italian, Spanish, Dutch, Portuguese, Japanese, Chinese (Simplified), Korean, Russian, Turkish, Polish, Arabic, Hindi, Thai, Vietnamese, Indonesian, Czech, Greek, Hungarian, Romanian, Swedish, Norwegian, Finnish, Danish, Bulgarian, Serbian, Croatian, Lithuanian, Estonian, Latvian, Slovenian, Slovak, Ukrainian, Hebrew, Malay, Filipino, Bengali, Tamil, Telugu, Kannada, Gujarati, Punjabi, Marathi, Urdu, Nepali, Sinhala, Khmer, Lao, Burmese, Mongolian, Fijian, Samoan, Tongan, Hawaiian, Maori, Tahitian	Patient	Yes/NA	Presenteeism	4 weeks	Yes, for presenteeism	No	Van der Burg et al., 2020 (69)
Sickness Presenteeism Scale-Nurse (SPS-N)	Generic	Self-reported	English, Turkish, Chinese	Patient	Yes/Yes	Presenteeism	1 month	No	No	Baris et al., 2023 (55)
Stanford Presenteeism Scale (SPS-6)	Generic	Self-reported	Chinese, English, Portuguese, Brazilian Portuguese, Italian, Dutch, Spanish, Persian	Patient	Yes/Yes	Presenteeism	1 month	No	No	Abdi et al., 2021 (50); Baldonedo-Mosteiro et al., 2020 (54); Bezzina et al., 2023 (56); Teoman and Harmanci Seren, 2022 (68); Ta-Thi and Chuang, 2022 (67); Seo et al., 2024 (64)
Stanford presenteeism scale-6 (shortened by two items) (Korean Version) (K-SPS-4)	Generic	Self-reported	Korean	Patient	Yes/Yes	Presenteeism	4 weeks	No	No	Seo et al., 2024 (64)
Stanford Presenteeism Scale-Short Form (Turkish version)	Generic	Self-reported	Turkish	Patient	Yes/Yes	Presenteeism	4 weeks	No	No	Teoman and Harmanci Seren, 2022 (68)
Work Ability Index (WAI)	Generic	Self-reported	English, Finnish, Spanish, Croatian, Thai, Malay, Persian, Swedish, Czech, Norwegian, Polish, Portuguese, French, German, Dutch, Italian, Russian, Romanian, Ukrainian, and more	Patient	Yes/NA	Presenteeism	Current work-ability as compared to their lifetime best	No	No	Van der Burg et al., 2020 (69)
Work Functioning Impairment Scale (WFun)	Generic	Self-reported	English, Japanese, Chinese	Patient	Yes/NA	Presenteeism	4 weeks, 1 week	Yes, for presenteeism	No	Ishimaru et al., 2020 (59)
Work Hoarse	Disease-specific	Self-reported	English	Patient	Yes/Yes	Presenteeism	7 days	Yes, for presenteeism	No	Zhu et al., 2019 (72)
Work Limitation Questionnaire (WLQ)	Generic	Self-reported	English +27 other languages	Patient	NA/Yes	Presenteeism, absenteeism	2 weeks	Yes, for absenteeism	No	Avance et al., 2024 (53)
Work Productivity and Activity Impairment Questionnaire (WPAI)	Generic	Self-reported	English, Spanish, Persian, Portuguese, Chinese, Czech, Dutch, French, German, Hungarian, Italian, Korean, Polish, Romanian, Russian, Ukrainian	Patient	Yes/Yes	Absenteeism, presenteeism, unpaid work	7 days	Yes, for absenteeism and presenteeism	No	D’Amico et al., 2020 (57); Ford et al., 2023 (58); Gelfand et al., 2020 (8); Ishimaru et al., 2020 (59); Wu et al., 2019 (70); Ta-Thi and Chuang, 2022 (66); Seyedrezazadeh et al., 2020 (65); Mamiya et al., 2025 (14); Kim et al., 2020 (60)
Work Productivity and Activity Impairment (WPAI) Specific Health Problem version 2 questionnaire	Disease-specific	Self-reported	More than 40 languages including English	Patient	NA/NA	Absenteeism, presenteeism, unpaid work	7 days	Yes, for absenteeism, presenteeism, and unpaid work	No	Ford et al., 2023 (58)
Work Productivity Impairments Questionnaire (for Psoriasis) (WPAI-PsO)	Disease-specific	Self-reported	English, Spanish	Patient	NA/NA	Absenteeism, presenteeism, unpaid work	7 days	Yes, for absenteeism and presenteeism	No	Wu et al., 2019 (70)
Work Role Functioning Questionnaire v2.0 (WRFQ) (+ Short Forms x2: WRFQ 5, WRFQ 10)	Generic	Self-reported	English, Dutch, Spanish, Brazilian Portuguese, Canadian French, Danish, Norwegian	Patient	Yes/Yes	Presenteeism	4 weeks	Yes, for presenteeism	No	Abma et al., 2019 (51)
World Health Organization Health and Performance Questionnaire (WHO-HPQ)	Generic	Self-reported	Chinese, English, German, Malay, Persian (Farsi), Traditional Chinese, Portuguese, Japanese	Patient	Yes/Yes	Presenteeism, absenteeism	4 weeks	Yes, for absenteeism and presenteeism	Supports HCA; Supports FCA	Kawakami et al., 2020 (61); Ta-Thi and Chuang, 2022 (66)

Abbreviations: FCA, friction cost approach; HCA, human capital approach; NA, not applicable.

The most frequently reported instruments were the Work Productivity and Activity Impairment Questionnaire (WPAI) (*n* = 9), the Stanford Presenteeism Scale (SPS-6) (*n* = 6), and the Institute for Medical Technology Assessment (iMTA) Productivity Cost Questionnaire (iPCQ), *n* = 4, often adapted or validated for specific populations (23–25). Instruments varied in scope: thirteen measured only presenteeism, four captured both presenteeism and absenteeism, and eight addressed all three components (presenteeism, absenteeism, and unpaid work). Twenty instruments allowed time-based quantification of productivity loss (e.g., hours worked at reduced capacity, days absent), while five relied on categorical or subjective assessments (e.g., Likert scales). Three instruments supported valuation of productivity loss using both the HCA and FCA: these were the iMTA iPCQ, its modified version for vocational rehabilitation (iPCQ-VR), and the World Health Organization Health and Performance Questionnaire (WHO-HPQ) (23;26;27). The three instruments were generic, self-administered for patients, were available in three or more languages, and demonstrated adequate validity and reliability. The iPCQ and iPCQ-VR allowed for quantification of all three components of productivity loss, whereas the WHO-HPQ captured presenteeism and absenteeism only. The remaining twenty-two instruments did not provide sufficient detail for valuation.

Supplementary Appendix 4 presents fifty-six instruments identified in previous reviews. Of these, ten overlapped with instruments we identified in our review, while the remaining forty-six were reported only in the previous reviews. Of these, twenty-six were generic instruments, and twenty were disease-specific. Five instruments were designed for interviewer administration, while the majority (*n* = 41) were for self-administration. Fifteen instruments were available in three or more languages, supporting broader cross-cultural use. Most instruments were designed for patients (*n* = 39), two intended for caregivers, three intended for parents, and two applicable to both patients and caregivers. The recall periods varied from as short as 1 day to as long as 2 years. Consistent with the instruments identified in our review, the majority of the additional instruments (*n* = 39) enabled quantification of at least one component of productivity loss. Of the forty-six instruments, twelve were suitable for monetary valuation using the HCA or FCA. Specifically, nine instruments supported the HCA, one instrument supported the FCA, and two instruments supported both the HCA and FCA. The remaining instruments did not provide sufficient detail for valuation using these approaches, such as inputs on duration, frequency, or wage-related information.

### Valuation methods

Eleven of the included studies explored valuation methods for productivity costs, primarily through comparisons of commonly used approaches or development of methodological frameworks (Table 3). Study designs included reviews (*n* = 4) and case studies applying methods in specific jurisdictions or to specific datasets (*n* = 7). Six studies examined both the HCA and the FCA within the same analysis, three focused exclusively on the FCA, and two explored compensation mechanisms (e.g., colleagues or temporary hires taking over tasks to offset lost work) and multiplier effects (e.g., the absence of one worker reducing the productivity of the entire team). Method choice often depended on data availability, such as market wage data or self-reported income, and on whether unpaid or informal work was considered relevant for the context (28). Two case studies applying both the HCA and FCA found consistently higher productivity costs with the HCA (29;30). Grega and Kolář concluded that the FCA is more appropriate, emphasizing that the HCA systematically overestimates productivity costs by assuming full employment, while the FCA better reflects labor market dynamics by limiting productivity losses to the friction period (31). Similarly, Setiawan et al. noted that HCA can lead to unrealistic overestimates, while the FCA is often constrained by a lack of friction period data, particularly in low- and middle-income countries (28).

**Table 3.** Valuation methods identified in peer-reviewed studies Table 3. long description.

Author, year	Country	Study design	Valuation methods	Conclusions and recommendations
Brouwer et al., 2023 (7)	Netherlands	Case study	Compensation mechanisms, Multiplier effects	Compensation mechanisms and multiplier effects are common and can significantly impact productivity losses from absenteeism and presenteeism, underscoring their relevance in such contexts
Grega and Kolář, 2021 (31)	Czech Republic	Review	HCA, FCA	FCA is more appropriate than HCA when calculating productivity costs when taking societal perspectives
Hanly et al., 2019 (29)	Ireland	Case study	HCA, FCA	Productivity loss should be reported in physical units, with cost estimates subjected to sensitivity analysis. If not feasible, use the HCA and national wages to value productivity loss when equity outweights effiency concerns
Hanly et al. 2020 (33)	Ireland	Case study	FCA	This study expanded FCA’s potential application beyond Europe and will support greater utilization of the FCA and wider inclusion of productivity costs in societal-based economic evaluations based on the use of widely available and updated key labor market variables in our selected countries
Hanly et al., 2023 (32)	Ireland	Case study	FCA	Two alternative and viable methods were developed to estimate country-specific friction periods. Method 1 BC (base-case) offers viable friction period estimates for countries where such data is lacking, while Method 2ALT (alternative) offers the advantage of using country-specific labor market data that can be updated over time. These approaches will enable researchers to apply the FCA to estimate the productivity cost of diseases across Europe from an employer’s perspective
Jiang et al., 2022 (73)	China	Review	FCA, HCA	A rising number of guidelines recommend including productivity loss in the base case or additional analyses of economic evaluation. We anticipate guidelines that are more suitable for Chinese practitioners and decision-makers that facilitate the advancement of research on productivity loss measurement and valuation
Krol et al., 2023 (13)	Netherlands	Systematic review	Multiplier effects, Compensation mechanisms	Compensation mechanisms and multiplier effects in productivity cost estimates should be included only when relevant evidence is available and validated methods exist, and otherwise should be limited to sensitivity analyses. Use or adapt existing instruments with validated modules, or develop new standardized tools. Consider correction factors adjusted for job, sector, and jurisdiction, and account for the costs of compensation mechanisms. Avoid double-counting with wages and explore broader firm-level or interfirm effects where relevant
Manipis et al., 2021 (34)	Australia	Case study	FCA	For diseases causing widespread absenteeism, adjustments for compensation mechanisms and multiplier effects may be appropriate. Team dynamics multipliers can be applied where disruption is significant. WTP estimates vary widely (e.g., 21-fold difference with versus without paid sick leave), highlighting the role of individual preferences and lost income. Including disutility of illness risks double-counting, since it may already be captured in both WTP values and QALYs. When combining WTP with the FCA, mismatches between employer and employee perspectives may arise. WTP estimates are also conditional on treatment uptake, which depends on factors such as age, education, income, and ability to pay
Manipis et al., 2025 (30)	Australia	Case study	FCA, HCA	The time taken to train an employee was the longest time component associated with replacing a worker in contrast to the traditional approach in the literature where a period of 28 days is normally applied to the vacancy duration to account for the recruitment decision and training. The high underemployment rate is consistent with the duration of the friction period being a shorter timeframe than the upper bound as reported in the literature (supporting the aforementioned theoretical relationship). Results also support compensation effects and highlight the need to adjust for the elasticity of time to labor productivity, especially for short absences. Internal labor reserves were more available for non-managers, while longer absences were harder to recover. In settings lacking vacancy data, recruiter surveys may offer an alternative for estimating friction period time and costs
Rissanen et al., 2021 (35)	Finland	Case study	HCA, FCA	Recommended to use of adjusted models of HCA and FCA whenever data are available, as they take into account the criticized hidden assumptions of the models and minimize the effects of possible biases
Setiawan et al., 2024 (28)	Australia	Systematic review	HCA, FCA	While the HCA is recommended in most guidelines, some argue that it is unrealistic and may lead to overestimation, as it does not consider that the work of the affected individuals could be replaced by others in a labor market. Conversely, not all countries have a friction period measure, hindering FCA’s implementation. Considerations for estimating the monetary value of productivity losses include whether time losses should be included for all types of populations (e.g., unpaid or informal work) and related inequity exacerbations, as well as data availability, such as whether market wages are available, and if they are deemed appropriate for the patient population. In addition, deciding whether it is feasible to collect self-reported income, including issues with recall bias, the cultural acceptability, and truthful response of income questions. If the population has a high proportion of informal workers, questions tailored to capture their income will need to be included, increasing the complexity of the survey. If the patient population includes those who are not in paid work, a decision will need to be made about if and how the monetary value will be estimated for their time losses. If the market wage and self-reported income methods are not feasible, then the willingness to pay method or macroeconomic measures may need to be used

Abbreviations: FCA, friction cost approach; HCA, human capital approach; QALY, quality-adjusted life years; WTP, willingness-to-pay.

Three studies refined or extended FCA methodologies (30;32;33). Hanly et al. proposed two strategies to estimate friction periods in countries lacking data: benchmarking Dutch vacancy stock and flow data, using routinely updated labor market data (33). Hanly et al. demonstrated that the FCA could be successfully extended beyond Europe using widely available labor statistics, such as applying Organisation for Economic Co-operation and Development (OECD) vacancy data to estimate the friction period in the US (32). Manipis et al. demonstrated that recruitment and training times drive replacement delays more than vacancy durations and emphasized underemployment and labor elasticity as key factors affecting friction periods (30).

Brouwer et al. and Krol et al. emphasized that compensation mechanisms (e.g., task reallocation, overtime) and multiplier effects (e.g., team-level spillovers) are common and can substantially influence productivity loss estimates (7;13). Manipis et al. examined the FCA in the context of mass absenteeism, suggesting that adjustments for team dynamics and multiplier effects may be justified (34). Rissanen et al. further recommended using adjusted variants of both the HCA and FCA to mitigate hidden assumptions, proposing modifications that account for unemployment rates, disability-free life expectancy, and work ability decline (35).

### Recommendations for measuring, valuing, and incorporating productivity losses in economic evaluations: environmental scan

The environmental scan identified ten relevant guidance documents addressing productivity costs in economic evaluations (Table 4). Most endorsed the societal perspective as the appropriate context for incorporating productivity costs. Canada’s National Advisory Committee on Immunization (NACI), CDA-AMC, and Institut National d’Excellence en Santé et en Services Sociaux (INESSS) recommended incorporating productivity in analyses adopting the societal perspective (36–38). Similarly, Germany’s Institute for Quality and Efficiency in Health Care (IQWiG) the cross-European Delphi consensus recommendations, and the New Dutch Guideline for Economic Evaluations in Healthcare recommended including productivity costs within societal perspectives (39–41). In contrast, the Belgian and Japanese guidelines recommended reporting productivity costs only in supplementary analyses or under certain assumptions, such as when the patient population is of working age and productivity impacts are expected (42;43). The US-based Value Assessment Framework proposed a different approach, recommending a “non-zero indirect” valuation even when direct evidence is unavailable (44).

**Table 4.** Results of the environmental scan of guidance on measuring, valuing, and incorporating productivity costs Table 4. long description.

Document	Country, year	Methods discussed	Recommendation
Canada’s Drug Agency Guidelines for the Economic Evaluation of Health Technologies: Canada (38)	Canada, 2017	Paid labor: HCA and FCA. Unpaid labor: Opportunity cost and replacement cost approaches	Paid labor: Use FCA for lost paid work time in the reference case (societal perspective) Unpaid labor: The opportunity cost approach is recommended. Researchers should be aware of and transparent about any equity implications, but ultimately the estimated opportunity cost should reflect the decision-maker’s equity position Leisure Time: Lost leisure time is not to be measured. Other methods may be used in nonreference case analyses
Belgian guidelines for economic evaluations and budget impact analyses (42)	Belgium, 2025	Paid labor: HCA and FCA	Productivity costs should not be included in the reference case but can be presented in a complementary analysis. The impact on productivity must be supported by evidence. Appropriate consideration should be given to the age distribution of the patient population and the consequent potential impact on productivity losses. In older, generally retired populations, it is inappropriate to account for productivity losses. Use HCA for short-term paid work. Use FCA for cases of long-term absence or death. The average friction period recommended for Belgium is 135 calendar days. No consensus exists on the best measurement instrument, so the suitability of the chosen tool should be verified
Consensus-based cross-European recommendations for the identification, measurement, and valuation of costs in health economic evaluations: a European Delphi study (39)	Netherlands, 2018	Paid labor: HCA and FCA Unpaid labor: opportunity costs (sex- and age-specific average wages).	Include the costs of absenteeism (paid and unpaid) and presenteeism in an economic evaluation from a societal perspective. Use FCA for paid work as the HCA is expected to lead to an overestimation of productivity losses. Use self-report questionnaires for measuring absenteeism and presenteeism. The use of sex- and age-specific average wages of unskilled labor is recommended for patient time and informal care
The BRAVER Roadmap to Broader Assessment of the Value of Health Interventions in the Asia-Pacific Region (45)	United Kingdom, 2025	Paid labor: HCA and FCA.	The majority of the expert advisory group agreed that productivity should be recognized and incorporated. However, experts recommended starting cautiously, using supplementary analyses and case studies to build evidence and test methods. Concerns include double-counting, equity implications (e.g. for children, elderly), and legal/policy limitations in some jurisdictions. The recognition of unpaid labor should also be considered to improve equitable outcomes.
Évaluation économique des interventions en santé et en services sociaux à l’Institut national d’excellence en santé et en services sociaux (INESSS) (37)	Canada, 2025	Productivity gains and losses: FCA Productivity cost of unpaid labor: opportunity cost approach	In cases where the societal perspective is adopted, productivity gains and losses must be valued using the FCA. The productivity cost of unpaid labor is valued using the opportunity cost approach. Lost leisure time should not be included in the cost assessment. The limitations associated with these analyses must be clarified
Guideline for Preparing Cost-Effectiveness Evaluation to the Central Social Insurance Medical Council (43)	Japan, 2022	Paid labor: HCA.	An analysis including productivity loss can be additionally performed on top of the base-case analysis. Productivity losses should be estimated using the HCA. The unit wage used should be the average wage across all industries, all ages, and both genders (or age group-specific). Actual employment status should be investigated, but if difficult, a 100% employment rate should be assumed for those 18+. Productivity losses for family members providing informal care are acceptable to include, using the same methods
Institut für Qualität und Wirtschaftlichkeit im Gesundheitswesen (IQWiG) – General Methods (40)	Germany, 2023	Paid labor: HCA and FCA. Unpaid labor: Opportunity cost (net wage) and replacement cost (gross wage) approaches.	Paid labor: Use the FCA from a societal perspective. HCA can be used in sensitivity analyses. Unpaid labor: value using the average net wage (opportunity cost approach). Replacement cost (gross wage) can be used in sensitivity analyses. Leisure Time: Exclude from the main analysis. Present in a sensitivity analysis if evidence allows, and it is not captured in the benefit parameter (e.g., QALY)
National Advisory Committee on Immunization (NACI) Guidelines for the Economic Evaluation of Vaccination Programs in Canada (36)	Canada, 2023	Paid labor: HCA and FCA. Unpaid labor: Replacement cost and opportunity cost approaches	For a societal perspective, productivity impacts should be systematically accounted for. Paid Work: Use the HCA in base-case analyses to support international comparability. Conduct a sensitivity analysis using a FCA. Unpaid Work: Value using the replacement cost approach as it aligns better with a societal perspective and avoids biases from income disparities. Lost leisure time should be excluded. Use average annual income and typical annual working hours across the Canadian labor force to estimate these losses. In most cases, there will be equity considerations related to whether and how productive time is valued. If productive time is differentially valued based on attributes such as age, gender, or health status, results of an economic evaluation could favor groups with the greatest income-earning potential and disadvantage other groups such as children who do not work or individuals with disabilities or severe health conditions that prevent them from holding high-income jobs. In these situations, researchers should conduct an additional sensitivity analysis using the average income and the average number of full-time hours worked for all Canadians based on Statistics Canada data
The New Dutch Guideline for Economic Evaluations in Healthcare: Taking the societal perspective to the next level (41)	The Netherlands, 2025	Paid labor: FCA	Within the societal perspective, include productivity costs using the FCA and costs of informal care
Institute for Clinical and Economic Review (ICER) – Value Assessment Framework (44)	United States, 2023	Nonzero indirect approach	Implement a nonzero indirect approach to include estimates of patient and caregiver productivity impacts in cost-effectiveness analyses conducted from a modified societal perspective, even when direct data are lacking. This approach provides a reasonable balance between informing on broader outcomes and encouraging more research. It involves valuing patient productivity loss during nonlife-extending periods and productivity gain (plus consumption costs) during life-extension, while caregiver time is approximated at 75% of the patient’s formal labor time lost

Abbreviations: FCA, friction cost approach; HCA, human capital approach; QALY, quality-adjusted life years.

#### Measuring and valuing paid labor

No guideline endorsed a specific measurement instrument. Belgium’s guideline recommended choosing methods based on appropriateness, while the cross-European Delphi consensus favored self-report questionnaires for measuring absenteeism and presenteeism (39;42). For the valuation of paid labor, several sources favored the FCA over the HCA. CDA-AMC recommended the FCA to reflect societal opportunity costs, but noted that if the HCA is used, losses should be valued with age- and sex-adjusted wages plus fringe benefits while recognizing HCA’s tendency to overstate losses (38). Similarly, IQWiG identified the FCA as preferred, restricting the HCA to sensitivity analyses to avoid double-counting productivity losses already captured in QALYs (40). The cross-European Delphi consensus recommendations also supported the FCA and cautioned against the HCA (39). In contrast, the Japanese guideline relied solely on the HCA, using average wages across industry, gender, and age categories, while assuming full employment for those aged 18 years and older when employment status is unknown (43). NACI recommended using a naïve HCA in base-case analyses for international comparability, alongside the FCA in sensitivity analyses, and specified wage sources from the Canadian labor force (36). Belgium’s national guidance supported the HCA for short-term productivity loss and the FCA for longer-term absences or mortality, setting a 135-day friction period (42).

#### Measuring and valuing unpaid labor

Unpaid labor was defined as volunteering, household production (e.g., cooking, cleaning, childcare), and informal caregiving (36;38;42). Guidance documents recommended valuing these losses using opportunity cost or replacement cost approaches. The Belgian guidelines advised measuring unpaid labor by estimating the time required if all patient activities were taken over by others, and reporting the incremental unpaid workdays (42). CDA-AMC and INESSS supported using the opportunity cost approach (38) (37), while IQWiG suggested using net wages (opportunity cost approach) in the base case and gross wages of an adequate replacement (replacement cost approach) in sensitivity analyses (40). NACI favored the replacement cost approach, emphasizing its relevance for equity because it avoids distortions related to wage disparities (36). The US Value Assessment Framework recommended approximating caregiver time at 75 percent of patient labor time lost (44). The cross-European Delphi consensus recommendations panel recommended valuing caregiving with sex- and age-specific average wages, emphasizing the importance of recognizing this cost category in older adults (39). The Japanese guideline recommended valuing caregiver productivity losses using the same HCA framework applied to paid labor (43).

#### Scope and equity considerations

Several guidelines (*n* = 4) explicitly advised excluding leisure time from productivity cost calculations. CDA-AMC and INESSS stated that it should not be measured (37;38), while IQWiG permitted inclusion only in sensitivity analyses when not already captured in utility measures (40). NACI similarly discouraged its inclusion to maintain consistency with pharmacoeconomic guidelines that prioritize labor-based valuations (36). Beyond leisure time, documents emphasized equity considerations in defining and valuing productivity. The BRAVER Roadmap cautioned that incorporating productivity costs may disadvantage populations who are not engaged in formal paid work, such as children and older adults, particularly when certain guidelines (e.g., Belgian guideline for economic evaluation) recommend against including losses for retired populations (42;45). Similarly, CDA-AMC highlighted that valuation methods for unpaid work, such as the opportunity cost approach, should reflect the decision-maker’s equity position (38). NACI explicitly warned that differentially valuing productive time by age, gender, or health status may bias evaluations in favor of those with the highest earning potential and recommended sensitivity analyses using population-average wages and working hours to mitigate this risk (36).

## Discussion

This scoping review and environmental scan synthesized peer-reviewed and gray literature on the measurement, valuation, and incorporation of patient and caregiver productivity costs in economic evaluations conducted from the societal perspective. We identified twenty-five instruments with wide variation in scope, recall period, and population, alongside additional instruments identified in prior reviews to provide a more comprehensive overview. Most enabled quantification, but only three (iPCQ, iPCQ-VR, WHO-HPQ) collected sufficient information to allow monetary valuation using the HCA or FCA (23;26;27). Caregiver productivity was especially underrepresented, with only two caregiver-specific instruments, neither suitable for valuation (46;47). This gap limits the ability of existing tools to generate data appropriate for economic evaluations. Instruments that can quantify components of productivity loss and support valuation through the HCA and FCA, such as the iPCQ and WHO-HPQ, are therefore likely the most convenient for future productivity loss measurement (23;27). However, their practical application also raises feasibility concerns: even where guidelines support inclusion, productivity costs are rarely incorporated in practice, with unpaid labor especially neglected due to methodological and data challenges (47). Further, measuring informal care time involves unresolved issues, such as incremental time, multitasking, and intangible tasks, that complicate data collection (48). In contexts where both patient and caregiver labor are affected, a single instrument may not capture the full scope of productivity loss, and the need to combine multiple tools could increase respondent burden. Balancing comprehensiveness and feasibility will be key to improving systematic inclusion of productivity costs in future evaluations.

We identified eleven studies that examined valuation methods and reviewed ten guidance documents from multiple jurisdictions to understand current methodological recommendations. Comparative work highlighted trade-offs between the HCA and FCA: the FCA yields more realistic estimates by restricting losses to the friction period (28;31), while the HCA, though internationally comparable, often overestimates losses by assuming continuous employment and ignoring compensation mechanisms (28;39). Reflecting this, agencies such as IQWiG and CDA have endorsed the FCA for paid work, with the HCA relegated to sensitivity analyses (38–40). Yet the FCA’s reliance on friction period data poses challenges in settings where such data are unavailable, leading some jurisdictions to recommend the HCA for the reference case analysis (32;36;43). Guidance on unpaid labor was inconsistent: some explicitly recommend the opportunity cost approach (e.g., CDA, INESSS), others support the replacement cost approach (e.g., NACI), (36–38), and IQWiG recommends opportunity cost in the reference case and replacement cost in sensitivity analyses (40). This lack of harmonization hinders international comparability and risks undervaluing caregiving in contexts with high informal care demands.

### Limitations and strengths

A key limitation of this study is the exclusion of primary economic evaluations from the review. As a result, we did not directly capture how productivity costs are currently applied in analyses. Second, while we reported on the validity and reliability of identified instruments when these were assessed in the included studies, we did not conduct a broader search for evidence on these properties, which would have required a separate strategy and was outside the scope of this review. Finally, we did not assess other important considerations, such as acceptability to respondents, recall accuracy, and instrument burden, which may also influence instrument selection. Our focus was instead on the instruments’ content and their suitability for quantifying and valuing productivity loss components for use in economic evaluations.

A key strength of this review is its broad scope, which encompassed the peer-reviewed literature and grey literature sources. By integrating methodological studies, systematic and narrative reviews, and national and international guidance documents, we captured not only the range of measurement instruments and valuation approaches reported in the literature but also the recommendations shaping current practice.

## Conclusions

While there are many instruments to measure productivity costs, few are suitable for valuation with the HCA and FCA, and even fewer are designed for caregivers. Concensus on valuation methods is lacking: the HCA is simple but overestimates, the FCA is conceptually stronger but data-intensive. Compensation mechanisms and multiplier effects have demonstrated significance in empirical studies, yet remain largely absent from international guidelines. Future research should prioritize the development and use of instruments that capture productivity in a valuation-ready format. Methodological work should refine friction period estimation in diverse labor markets and consider correction factors for compensation and multiplier effects. At the policy level, greater alignment across HTA bodies on core principles, such as when productivity costs are included in the base case analysis and preferred valuation methods, would substantially strengthen comparability and transferability of evaluations conducted from the societal perspective. Finally, future research should assess the extent to which current economic evaluations adhere to existing guidance on the incorporation of productivity costs, identifying specific areas where practice diverges from recommended standards to ensure methodological rigor is not lost in application.

## Supporting information

10.1017/S0266462326103882.sm001 Yeretzian et al. supplementary material Yeretzian et al. supplementary material

## Supplementary material

The supplementary material for this article can be found at http://doi.org/10.1017/S0266462326103882.

## Long descriptions

### Figure 1. Long description

A total of 9,292 records were identified from Embase and MEDLINE (8,607 records) and EconLit (685 records). After the removal of 93 duplicate records, 9,199 records were screened, and 9,055 were excluded. Full texts were sought for 144 reports, all of which were retrieved. After restricting eligibility to studies published between 2019 and the present, 49 reports remained and were assessed for eligibility. Nine reports were excluded because they were not measurement tools (n = 8) or did not measure productivity loss (n = 1). A total of 40 studies were included in the review.

Navigate back to Figure 1..

### Table 1. Long description

The studies are divided into two sections. The first and larger section contains studies examining productivity cost measurement instruments. These studies include instrument development, validation, adaptation, methodological evaluations, and evidence syntheses. A wide range of productivity instruments are represented. Several studies adapted or validated existing instruments for use in different languages and countries. The second section contains studies examining productivity cost valuation methods. These studies primarily evaluate the human capital approach (HCA) and friction cost approach (FCA), with some also assessing multiplier effects and compensation mechanisms.

Navigate back to Table 1..

### Table 2. Long description

Most instruments were designed for patients and measured presenteeism, absenteeism, unpaid work, or a combination of these outcomes. Only the iPCQ, iPCQ-VR, and WHO-HPQ were identified as suitable for valuation using both the Human Capital Approach and Friction Cost Approach.

Navigate back to Table 2..

### Table 3. Long description

The studies evaluated the Human Capital Approach (HCA), Friction Cost Approach (FCA), compensation mechanisms, and multiplier effects. Several studies compared the HCA and FCA, often concluding that the HCA produces higher productivity cost estimates, while the FCA better reflects labor market dynamics. Other studies highlighted the importance of accounting for compensation mechanisms, multiplier effects, and country-specific labor market conditions when estimating productivity costs.

Navigate back to Table 3..

### Table 4. Long description

Most guidelines recommend considering productivity costs when analyses adopt a societal perspective. Recommendations varied regarding the preferred valuation approach, with several guidelines favouring the Friction Cost Approach (FCA), others recommending the Human Capital Approach (HCA), and some advising the use of both approaches in base-case or sensitivity analyses. The table also summarizes guidance on valuing unpaid labor and highlights equity considerations related to productivity cost estimation.

Navigate back to Table 4..
