# Supplementary material for: Measuring, valuing, and incorporating patient and caregiver productivity costs in economic evaluations: a scoping review and environmental scan
Source: Int J Technol Assess Health Care. 2026 Jun 26;42(1):e64. doi: 10.1017/S0266462326103882 (PMC13440446; doi:10.1017/S0266462326103882)
Supplement: Supplementary file 1 [file S0266462326103882sup001.docx]

**APPENDIX**

**Appendix 1: Preferred Reporting Items for Systematic reviews and Meta-Analyses extension for Scoping Reviews (PRISMA-ScR) Checklist**

| **SECTION** | **ITEM** | **PRISMA-ScR CHECKLIST ITEM** | **REPORTED ON PAGE #** |
| --- | --- | --- | --- |
| **TITLE** | | | |
| Title | 1 | Identify the report as a scoping review. | 1 |
| **ABSTRACT** | | | |
| Structured summary | 2 | Provide a structured summary that includes (as applicable): background, objectives, eligibility criteria, sources of evidence, charting methods, results, and conclusions that relate to the review questions and objectives. | 2 |
| **INTRODUCTION** | | | |
| Rationale | 3 | Describe the rationale for the review in the context of what is already known. Explain why the review questions/objectives lend themselves to a scoping review approach. | 3-4 |
| Objectives | 4 | Provide an explicit statement of the questions and objectives being addressed with reference to their key elements (e.g., population or participants, concepts, and context) or other relevant key elements used to conceptualize the review questions and/or objectives. | 4 |
| **METHODS** | | | |
| Protocol and registration | 5 | Indicate whether a review protocol exists; state if and where it can be accessed (e.g., a Web address); and if available, provide registration information, including the registration number. | 5 |
| Eligibility criteria | 6 | Specify characteristics of the sources of evidence used as eligibility criteria (e.g., years considered, language, and publication status), and provide a rationale. | 6 |
| Information sources* | 7 | Describe all information sources in the search (e.g., databases with dates of coverage and contact with authors to identify additional sources), as well as the date the most recent search was executed. | 6 |
| Search | 8 | Present the full electronic search strategy for at least 1 database, including any limits used, such that it could be repeated. | 36-38 |
| Selection of sources of evidence† | 9 | State the process for selecting sources of evidence (i.e., screening and eligibility) included in the scoping review. | 6 |
| Data charting process‡ | 10 | Describe the methods of charting data from the included sources of evidence (e.g., calibrated forms or forms that have been tested by the team before their use, and whether data charting was done independently or in duplicate) and any processes for obtaining and confirming data from investigators. | 7 |
| Data items | 11 | List and define all variables for which data were sought and any assumptions and simplifications made. | 7 |
| Critical appraisal of individual sources of evidence§ | 12 | If done, provide a rationale for conducting a critical appraisal of included sources of evidence; describe the methods used and how this information was used in any data synthesis (if appropriate). | NA |
| Synthesis of results | 13 | Describe the methods of handling and summarizing the data that were charted. | 9 |
| **RESULTS** | | | |
| Selection of sources of evidence | 14 | Give numbers of sources of evidence screened, assessed for eligibility, and included in the review, with reasons for exclusions at each stage, ideally using a flow diagram. | 9-10 |
| Characteristics of sources of evidence | 15 | For each source of evidence, present characteristics for which data were charted and provide the citations. | 9-11 |
| Critical appraisal within sources of evidence | 16 | If done, present data on critical appraisal of included sources of evidence (see item 12). | NA |
| Results of individual sources of evidence | 17 | For each included source of evidence, present the relevant data that were charted that relate to the review questions and objectives. | 10-12 |
| Synthesis of results | 18 | Summarize and/or present the charting results as they relate to the review questions and objectives. | 10-12 |
| **DISCUSSION** | | | |
| Summary of evidence | 19 | Summarize the main results (including an overview of concepts, themes, and types of evidence available), link to the review questions and objectives, and consider the relevance to key groups. | 15-16 |
| Limitations | 20 | Discuss the limitations of the scoping review process. | 16-17 |
| Conclusions | 21 | Provide a general interpretation of the results with respect to the review questions and objectives, as well as potential implications and/or next steps. | 17-18 |
| **FUNDING** | | | |
| Funding | 22 | Describe sources of funding for the included sources of evidence, as well as sources of funding for the scoping review. Describe the role of the funders of the scoping review. | 18 |

**Appendix 2: Search Strategy**

**Databases:** Embase; Ovid MEDLINE

**Date run:** July 09, 2025

| # | Searches |
| --- | --- |
| 1 | Absenteeism/ or Presenteeism/ or Sick Leave/ |
| 2 | (Unemployment/ or Employment/ or Return to Work/ or Work/ or *Income/ or *"Salaries and Fringe Benefits"/) and (Caregivers/ or exp Patients/ or (caregiv* or care-giv* or patient* or inpatient* or outpatient*).ti,kf.) |
| 3 | (presenteeism* or absenteeism*).ti,kf. |
| 4 | (presenteeism* or absenteeism*).ab. /freq=3 |
| 5 | (productivity adj2 (cost* or decreas* or gain* or loss* or reduc* or work* or measure or measures or measurement or evaluat*)).ti,ab,kf. |
| 6 | (productivity adj4 (illness or treatment or disability or death)).ti,ab,kf. |
| 7 | (production adj2 (cost$ or valuation$)).ab. /freq=2 |
| 8 | ((spill-over or spillover) adj2 (family or caregiv* or care-giv* or patient*)).ti,ab,kf. |
| 9 | ((compensate* or informal or paid or uncompensate* or un-compensate* or unpaid or un-paid) adj2 (care or caregiv* or care-giv* or labor or labour or production or work)).ti,kf. |
| 10 | ((paid or unpaid or un-compensate* or uncompensated* or compensate*) adj2 (activity or activities or hour* or time)).ti,ab,kf. |
| 11 | ((caregiv* or care-giv* or patient*) adj2 burden*).ti,kf. |
| 12 | ((caregiv* or care-giv* or patient*) adj4 productivity).ti,kf. |
| 13 | or/1-12 |
| 14 | *Psychometrics/ or *Reproducibility of Results/ or *Self Report/ or *"Surveys and Questionnaires"/ or *Efficiency, Organizational/ or *Efficiency/ |
| 15 | ((instrument* or measure$ or measurement$ or measuring or calculat* or index* or indice$ or indicie* or inventories or inventory or questionnaire$ or scale$ or survey$) adj3 (absenteeism or presenteeism or productivity or production* or labour or labor or work)).ab. /freq=3 |
| 16 | ((instrument* or measure$ or measurement$ or measuring or calculat* or index* or indice$ or indicie* or inventories or inventory or questionnaire$ or scale$ or survey$) adj2 (absenteeism or presenteeism or productivity or production* or labour or labor or work)).ti. |
| 17 | (productivity adj2 (instrument* or measure$ or measurement$)).ab. |
| 18 | ((economic or valuation* or value$ or perspective* or cost* or benefit* or gain* or loss* or viewpoint* or view-point* or impact*) adj4 (assessment* or production or productivity or work* or caregiv* or care-giv* or labor or labour)).ti. |
| 19 | ((economic or valuation* or value$ or perspective* or cost* or benefit* or gain* or loss* or viewpoint* or view-point* or impact*) adj2 (assessment* or production or productivity or work* or caregiv* or care-giv* or labor or labour)).ab. /freq=2 |
| 20 | ((economic or valuation* or value$ or perspective* or cost* or benefit* or gain* or loss* or viewpoint* or view-point* or impact*) adj2 (assessment* or production or productivity or work* or caregiv* or care-giv* or labor or labour)).kf. |
| 21 | or/14-20 |
| 22 | 13 and 21 |
| 23 | *Absenteeism/ec, mt or *Presenteeism/ec, mt or *Sick Leave/ec, mt |
| 24 | 22 or 23 |
| 25 | 24 use medall |
| 26 | *productivity/ or *absenteeism/ or *presenteeism/ |
| 27 | (exp *employment/ or *unemployment/ or *medical leave/ or *work/ or *income/) and (*caregiver burden/ or *caregiver/ or *caregiver support/ or *patient/) |
| 28 | (presenteeism* or absenteeism*).ti,kf. |
| 29 | (presenteeism* or absenteeism*).ab. /freq=3 |
| 30 | (productivity adj2 (cost* or decreas* or gain* or loss* or reduc* or work* or measure or measures or measurement or evaluat*)).ti,ab,kf. |
| 31 | (productivity adj4 (illness or treatment or disability or death)).ti,ab,kf. |
| 32 | (production adj2 (cost$ or valuation$)).ab. /freq=2 |
| 33 | ((spill-over or spillover) adj2 (family or caregiv* or care-giv* or patient*)).ti,ab,kf. |
| 34 | ((compensate* or informal or paid or uncompensate* or un-compensate* or unpaid or un-paid) adj2 (care or caregiv* or care-giv* or labor or labour or production or work)).ti,kf. |
| 35 | ((paid or unpaid or un-compensate* or uncompensated* or compensate*) adj2 (activity or activities or hour* or time)).ti,ab,kf. |
| 36 | ((caregiv* or care-giv* or patient*) adj2 burden*).ti,kf. |
| 37 | ((caregiv* or care-giv* or patient*) adj4 productivity).ti,kf. |
| 38 | or/26-37 |
| 39 | *psychometry/ or exp *health survey/ or exp *questionnaire/ or *reproducibility/ |
| 40 | ((instrument* or measure$ or measurement$ or measuring or calculat* or index* or indice$ or indicie* or inventories or inventory or questionnaire$ or scale$ or survey$) adj3 (absenteeism or presenteeism or productivity or production* or labour or labor or work)).ab. /freq=3 |
| 41 | ((instrument* or measure$ or measurement$ or measuring or calculat* or index* or indice$ or indicie* or inventories or inventory or questionnaire$ or scale$ or survey$) adj2 (absenteeism or presenteeism or productivity or production* or labour or labor or work)).ti. |
| 42 | (productivity adj2 (instrument* or measure$ or measurement$ or measuring)).ab. |
| 43 | ((economic or valuation* or value$ or perspective* or cost* or benefit* or gain* or loss* or viewpoint* or view-point* or impact*) adj4 (assessment* or production or productivity or work* or caregiv* or care-giv* or labor or labour)).ti. |
| 44 | ((economic or valuation* or value$ or perspective* or cost* or benefit* or gain* or loss* or viewpoint* or view-point* or impact*) adj2 (assessment* or production or productivity or work* or caregiv* or care-giv* or labor or labour)).ab. /freq=2 |
| 45 | ((economic or valuation* or value$ or perspective* or cost* or benefit* or gain* or loss* or viewpoint* or view-point* or impact*) adj2 (assessment* or production or productivity or work* or caregiv* or care-giv* or labor or labour)).kf. |
| 46 | or/39-45 |
| 47 | 38 and 46 |
| 48 | 47 use oemezd |
| 49 | 25 or 48 |
| 50 | (clinical trial or conference review or conference abstract or comment or newspaper article or editorial or letter or note).pt. |
| 51 | 49 not 50 |
| 52 | limit 51 to yr="1996 -Current" |
| 53 | remove duplicates from 52 |

**Database: EconLit** (EBSCOhost)

**Date run:** July 17, 2025

| # | Searches |
| --- | --- |
| 1 | TI (presenteeism OR absenteeism) |
| 2 | AB (presenteeism OR absenteeism) |
| 3 | TI ((productivity) N2 (cost# OR decrease# OR gain# OR loss# OR reduce# OR reduction# OR work OR measure OR measures OR measurement OR evaluate# OR evaluation#)) |
| 4 | TI ((productivity) N4 (illness OR treatment OR disability OR death)) |
| 5 | TI ((spill-over OR spillover) N2 (family OR caregiver# OR care-giver# OR patient#)) |
| 6 | TI ((compensate# OR informal OR paid OR uncompensated# OR un-compensate# OR unpaid OR un-paid) N2 (care OR caregiver# OR care-giver# OR labor OR labour OR production OR work)) |
| 7 | TI ((paid OR unpaid OR un-compensate# OR uncompensated# OR compensate#) N2 (activity OR activities OR hour# OR time)) |
| 8 | TI ((caregiver# OR care-giver# OR patient#) N2 burden#) |
| 9 | TI ((caregiver# OR care-giver# OR patient#) N4 productivity) |
| 10 | or/1-9 [*Set 1*] |
| 11 | TI ((instrument# OR instrumentation OR measure# OR measurement# OR measuring OR calculate# OR calculation# OR index OR indice# OR indicies OR inventories OR inventory OR questionnaire# OR scale# OR survey#) N3 (absenteeism OR presenteeism OR productivity OR production OR labour OR labor OR work)) |
| 12 | AB (productivity N2 (instrument# OR instrumentation# OR measure# OR measurement#)) |
| 13 | TI ((economic OR valuation# OR value# OR perspective# OR cost# OR benefit# OR gain# OR loss OR losses OR viewpoint# OR view-point# OR impact#) N2 (assessment# OR production OR productivity OR work OR caregiver# OR care-giver# OR labor OR labour)) |
| 14 | or/11-13 [*Set 2*] |
| 15 | 10 and 14 [*Set 1 and Set 2*] |
| 16 | Limit 15 to 1999-present |

**Appendix 3: Sources Searched in the Environmental Scan**

| **Agency/Source** | **Jurisdiction** | **URL** |
| --- | --- | --- |
| Institute of Health Economics | Canada | <http://www.ihe.ca/index.php?/publications> |
| National Health Service UK | United Kingdom | <http://www.england.nhs.uk/> |
| Austrian Institute for Health Technology Assessment | Austria | <http://eprints.aihta.at/> |
| Institute of Technology Assessment | Austria | <https://www.oeaw.ac.at/en/ita/search-for-publications> |
| Belgian Health Care Knowledge Centre | Belgium | <https://www.kce.fgov.be/en/publications/all-reports-0> |
| CEDIT Recommendations and Reports | France | <http://cedit.aphp.fr/cedit-hospital-based-hta-agency/recommendations-reports/> |
| Haute Autorité de santé | France | <https://www.has-sante.fr/> |
| Institute for Quality and Efficiency in Health Care (IQWIG) reports | Germany | [https://www.iqwig.de/en](https://www.iqwig.de/en/projects/projects-results/#searchQuery=query=*&page=1&rows=10&sortBy=score&sortOrder=desc&facet.filter.language=en) |
| European Network for Health Technology Assessment (EUnetHTA) | General | <https://tools.eunethta.be/index.html> |
| International Network of Agencies for Health Technology Assessment | General | <http://www.inahta.org/publications/> |
| The International Network of Agencies for Health Technology Assessment (INAHTA) database | General | <https://database.inahta.org/> |
| Health Evidence Network | General | https://www.who.int/europe/groups/health-evidence-network-(hen) |
| Health Information and Quality Authority | Ireland | <https://www.hiqa.ie/reports-and-publications/health-technology-assessments> |
| Irish Health Repository | Ireland | <http://www.lenus.ie/hse/> |
| Institute of Health Carlos III | Spain | <https://www.isciii.es/en/> |
| The Commonwealth Fund | United States | <http://www.commonwealthfund.org/topics> |
| Monash Health. Centre for Clinical Effectiveness | Australia | <http://monashhealth.org/health-professionals/cce/cce-publications/> |
| Queensland Government (Australia). Health Technology Reference Group | Australia | <https://www.health.gov.au/our-work/hta-review/review-analysis-papers> |
| Sax Institute | Australia | <https://www.saxinstitute.org.au/resource-hub/?_evidence_check=evidence_check> |
| Alberta Health | Canada | <https://www.alberta.ca/health-evidence-reviews.aspx> |
| British Columbia Ministry of Health | Canada | <https://www2.gov.bc.ca/gov/content/health/about-bc-s-health-care-system/partners/health-authorities/bc-health-technology-assessment/health-technology-assessments#current> |
| Canada’s Drug Agency (CDA-AMC) | Canada | <https://www.cda-amc.ca/> |
| l'Institut national de santé publique du Québec (INSPQ) | Canada | <https://www.inspq.qc.ca/en/publications> |
| Health Quality Council of Alberta | Canada | <https://hqca.ca/reports-library/> |
| Health Quality Ontario | Canada | <http://www.hqontario.ca/Evidence-to-Improve-Care/Health-Technology-Assessment> |
| Manitoba Centre for Health Policy | Canada | <http://mchp-appserv.cpe.umanitoba.ca/deliverablesList.html> |
| Contextualized Health Research Synthesis Program (CHRSP) projects | Canada | <https://www.mun.ca/nlcahr/chrsp/completed-chrsp-projects/> |
| O'Brien Institute for Public Health | Canada | <https://obrieniph.ucalgary.ca/groups/health-technology-assessment-unit/projects> |
| Ottawa Hospital Research Institute | Canada | <https://www.ohri.ca/OurResearch/default.asp> |
| Programs for Assessment of Technology in Health (PATH) | Canada | <https://www.path-hta.com/> |
| Technology Assessment at SickKids (TASK) | Canada | <http://lab.research.sickkids.ca/task/reports-theses/> |
| L'Institut national d'excellence en santé et en services sociaux (INESSS) | Canada | <https://www.inesss.qc.ca/en/home.html> |
| Centre for Health Services and Policy Research | Canada | <http://chspr.ubc.ca/publications/> |
| Healthcare Improvement Scotland | United Kingdom | [http://www.healthcareimprovementscotland.org](http://www.healthcareimprovementscotland.org/) |
| NIHR Evaluation, Trials and Studies Coordinating Centre | United Kingdom | <https://www.journalslibrary.nihr.ac.uk/> |
| National Institute for Health Research | United Kingdom | <http://www.io.nihr.ac.uk/> |
| The National Institute for Health and Care Excellence (NICE) | United Kingdom | <http://www.nice.org.uk/> |
| Agency for Healthcare Research and Quality (AHRQ) | United States | <https://www.ahrq.gov/research/findings/ta/index.html> |
| Institute for Clinical and Economic Review (ICER) | United States | <https://icer.org/explore-our-research/> |
| The Netherlands Organisation for Health Research and Development | Netherlands | <https://www.zonmw.nl/en> |
| Pharmaceutical Management Agency (Pharmac) | New Zealand | <https://www.pharmac.govt.nz/> |
| National Advisory Committee on Immunization (NACI) | Canada | <https://www.canada.ca/en/public-health/services/immunization/national-advisory-committee-on-immunization-naci/methods-process.html> |
| National Health Care Institute | Netherlands | [Home \| National Health Care Institute](https://english.zorginstituutnederland.nl/) |
| Guidelines International Network | General | <https://g-i-n.net/international-guidelines-library/> |
| ECRI Guidelines Trust | General | <https://home.ecri.org/pages/ecri-guidelines-trust> |
| Centre for Reviews and Dissemination | Canada | <https://www.crd.york.ac.uk/CRDWeb/> |
| Pharmaceutical Benefits Scheme | Australia | <http://www.pbs.gov.au/browse/medicine-listing> |
| Centre for Health Economics and Policy Analysis. Publications database | Canada | https://chepa.mcmaster.ca/ |
| Toronto Health Economics and Technology Assessment Collaborative | Canada | [http://theta.utoronto.ca](http://theta.utoronto.ca/) |
| National Centre for Pharmacoeconomics | Ireland | <http://www.ncpe.ie/pharmacoeconomic-evaluations/> |
| Office of Health Economics | United Kingdom | <https://www.ohe.org/publications> |
| Health Economics Research Unit | United Kingdom | <http://www.abdn.ac.uk/heru/publications-page-687.php> |
| AHRQ National Quality Measures Clearinghouse | United States | <https://www.ahrq.gov/gam/index.html> |
| RePEc IDEAS database | General | https://ideas.repec.org/ |

**Appendix 4: Additional Instruments from Included Reviews**

| **Instrument Name** | **Generic or Disease-Specific** | **Mode of Administration** | | **Languages Available** | **Population** | **Validity/ Reliability** | | | **Productivity Cost Components Captured** | **Recall Period** | | **Does it allow for quantification?** | | **Valuation Suitability** | | **Source** | |  |  |
| --- | --- | --- | --- | --- | --- | --- | --- | --- | --- | --- | --- | --- | --- | --- | --- | --- | --- | --- | --- |
| **Instruments identified in other reviews captured in our study** | | | | | | | | | | | | | | | | | | |  |
| Work Productivity and Activity Impairment Questionnaire (WPAI) | Generic | Self-reported | English, Spanish, Persian, Portuguese, Chinese, Czech, Dutch, French, German, Hungarian, Italian, Korean, Polish, Romanian, Russian, Ukrainian | | Patient | | Yes/Yes | Absenteeism, presenteeism, unpaid work | | | 7 days | | Yes, for absenteeism and presenteeism | | No | | Hubens et al. 2021(1); Mamiya et al. 2025(2); Zhang et al. 2023(3) | | |
| iMTA productivity cost questionnaire (iPCQ) | Generic | Self-reported | Arabic, English, Dutch, Czech, Danish, Finnish, French, German, Hungarian, Italian, Korean, Norwegian, Polish, Portuguese, Romanian, Spanish, Turkish | | Patient | | Yes/Yes | Absenteeism, presenteeism, unpaid work | | | 4 weeks | | Yes, for absenteeism, presenteeism, and unpaid work | | Supports HCA; Supports FCA | | Hubens et al. 2021(1); Mamiya et al. 2025(2) | | |
| World Health Organization Health and Performance Questionnaire (WHO-HPQ) | Generic | Self-reported | Chinese, English, German, Malay, Persian (Farsi), Traditional Chinese, Portuguese, Japanese | | Patient | | Yes/NA | Presenteeism, absenteeism | | | 4 weeks | | Yes, for absenteeism and presenteeism | | Supports HCA; Supports FCA | | Hubens et al. 2021(1); Mamiya et al. 2025(2); Zhang et al. 2023(3) | | |
| The Migraine Disability Assessment Score (MIDAS) | Disease-specific | Self-reported | English | | Patient | | Yes/NA | Presenteeism, absenteeism | | | 3 months | | Yes, for absenteeism, presenteeism, and unpaid work | | No | | Hubens et al. 2021(1); Mamiya et al. 2025(2) | | |
| Work Limitation Questionnaire (WLQ) | Generic | Self-reported | English + 27 other languages | | Patient | | NA/NA | Presenteeism, absenteeism | | | 2 weeks | | Yes, for absenteeism | | No | | Hubens et al. 2021(1); Zhang et al. 2023(3) | | |
| Quantity and Quality (QQ) method | Generic | Self-reported | English, German, French, Italian, Spanish, Dutch, Portuguese, Japanese, Chinese (Simplified), Korean, Russian, Turkish, Polish, Arabic, Hindi, Thai, Vietnamese, Indonesian, Czech, Greek, Hungarian, Romanian, Swedish, Norwegian, Finnish, Danish, Bulgarian, Serbian, Croatian, Lithuanian, Estonian, Latvian, Slovenian, Slovak, Ukrainian, Hebrew, Malay, Filipino, Bengali, Tamil, Telugu, Kannada, Gujarati, Punjabi, Marathi, Urdu, Nepali, Sinhala, Khmer, Lao, Burmese, Mongolian, Fijian, Samoan, Tongan, Hawaiian, Maori, Tahitian | | Patient | | NA/NA | Presenteeism | | | 4 weeks | | Yes, for presenteeism | | No | | Hubens et al. 2021(1); Zhang et al. 2023(3) | | |
| Valuation of Lost Productivity (VOLP) Questionnaire | Generic | Self-reported | English, Dutch, French, German, Italian, Spanish, Arabic, Romanian, Polish, Hungarian, Norwegian, and Russian | | Caregiver | | NA/NA | Absenteeism, presenteeism, unpaid work | | | 3 months (absenteeism) and also 7 days (presenteeism) | | Yes, for absenteeism, presenteeism, and unpaid work | | No | | Hubens et al. 2021(1) | | |
| Work Productivity and Activity Impairment (WPAI) Specific Health Problem version 2 questionnaire | Disease-specific | Self-reported | English, Spanish | | Patient | | Yes/Yes | Absenteeism, presenteeism, unpaid work | | | 7 days | | Yes, for absenteeism and presenteeism | | No | | Hubens et al. 2021(1) | | |
| Stanford Presenteeism Scale (SPS-6) | Generic | Self-reported | Chinese, English, Portuguese, Brazilian Portuguese, Italian, Dutch, Spanish, Persian | | Patient | | NA/NA | Presenteeism | | | 1 month | | No | | No | | Hubens et al. 2021(1) | | |
| Work Hoarse (VRS-8) | Disease-specific | Self-reported | English | | Patient | | Yes/Yes | Presenteeism | | | 7 days | | Yes, for presenteeism | | No | | Hubens et al. 2021(1) | | |
| **Instruments identified in other reviews not captured in our study** | | | | | | | | | | | | | | | | | | |  |
| Bespoke questionnaire | Generic | Self-report | | Language questionnaire was created in | Patient | NA/NA | | | Work-time loss | NA | | No | | No | | Mamiya et al. 2025(2) | |  |  |
| Client Service Receipt Inventory (CSRI) | Generic | Self-report | | Danish, English, Italian, Dutch, Spanish, German, Portuguese (Brazilian) | Patient and caregiver | Yes/NA | | | Work-time loss | NA | | Yes, for work-time loss | | Yes, for opportunity costs approach | | Mamiya et al. 2025(2) | |  |  |
| Client Service Receipt Inventory modifed version 8 (CSRI-modified) | Generic | Self-report | | Danish, English, Italian, Dutch, Spanish, German, Portuguese (Brazilian) | Caregiver | NA/NA | | | Work-time loss | 3 months | | Yes, for work-time loss | | Yes, for opportunity costs approach | | Hubens et al. 2021(1) | |  |  |
| Client Sociodemographic and Service Receipt Inventory | Generic | Self-report | | English, German, Dutch, Italian, Spanish, Portuguese | Patient | Yes/NA | | | Absenteeism | 3 - 12 months | | Yes for absenteeism | | Yes, for HCA | | Mamiya et al. 2025(2) | |  |  |
| Cost Interview | Disease-specific | Interview | | German | Patient | NA/NA | | | Absenteeism | 12 months | | Yes, for absenteeism | | Yes, for HCA | | Hubens et al. 2021(1) | |  |  |
| Economic Implications of Psoriasis Patient Questionnaire | Disease-specific | Self-report | | English | Patient | NA/NA | | | Absenteeism, presenteeism, and unpaid work | 1 week | | Yes for absenteeism, presenteeism, and unpaid work | | Yes, for HCA | | Zhang et al. 2023(3) | |  |  |
| Endicott Work Productivity Scale (EWPS) | Generic | Self-report | | English and Turkish | Patient | NA/NA | | | Absenteeism and presenteeism | 1 week | | Yes, for absenteeism | | No | | Hubens et al. 2021(1) | |  |  |
| Family Cost Survey (FCS, version 1.0) | Disease-specific | Self-report | | English | Parent | NA/NA | | | Absenteeism | NA | | Yes, for absenteeism | | No | | Hubens et al. 2021(1) | |  |  |
| Headache-Attributed Lost Time Indices 30 (HALT-30 Index) | Disease-specific | Self-report | | English, Dutch, French, German, Italian, Portuguese, and Spanish | Patient | NA/NA | | | Absenteeism, presenteeism, and unpaid work | 30 days | | Yes, for absenteeism, presenteeism, and unpaid work | | No | | Hubens et al. 2021(1) | |  |  |
| Headache-Attributed Lost Time Indices 7/30 (HALT-7/30 Index) | Disease-specific | Self-report | | English, Dutch, French, German, Italian, Portuguese, and Spanish | Patient | NA/NA | | | Presenteeism and absenteeism | 7 and 30 days | | Yes, for absenteeism and presenteeism | | No | | Hubens et al. 2021(1) | |  |  |
| Headache-Attributed Lost Time Indices 90 (HALT-90 Index) | Disease-specific | Self-report | | English, Dutch, French, German, Italian, Portuguese, and Spanish | Patient | NA/NA | | | Absenteeism, presenteeism, and unpaid work | 90 days | | Yes, for presenteeism and absenteeism | | No | | Hubens et al. 2021(1) | |  |  |
| Health and Labour Questionnaire (HLQ) | Generic | Self-report | | Dutch, English | Patient | Yes/NA | | | Absenteeism, presenteeism, and unpaid work | 2 weeks | | Yes for Absenteeism, presenteeism, and unpaid work | | Yes for FCA and HCA | | Hubens et al. 2021(1) | |  |  |
| Health and Labour Questionnaire-Short Form (SF-HLQ) | Generic | Self-report | | Dutch, English | Patient | NA/NA | | | Absenteeism, presenteeism, and unpaid work | 1 month | | Yes for Absenteeism, presenteeism, and unpaid work | | Yes for FCA and HCA | | Hubens et al. 2021(1) | |  |  |
| Health and Performance Questionnaire (HPQ, 7-day clinical trial version) | Generic | Self-report | | English, Spanish | Patient | NA/NA | | | Absenteeism and presenteeism | 7 days | | Yes for absenteeism and presenteeism | | No | | Hubens et al. 2021(1) | |  |  |
| Health and Retirement Survery | Generic | Self-report | | English, Spanish, Mandarin Chinese, French, German, Italian, Greek, Dutch, Swedish, Danish, Finnish, Polish, Czech, Slovenian, Estonian, Hebrew, Portuguese, Korean, Japanese, Indonesian, Turkish | Patient | NA/NA | | | Absenteeism and unpaid work | 2 years | | Yes for absenteeism and unpaid work | | Yes, for HCA | | Zhang et al. 2023(3) | |  |  |
| Health and Work Questionnaire (HWQ) | Generic | Self-report | | English | Patient | NA/NA | | | Presenteeism | 1 week | | Yes, for presenteeism | | No | | Hubens et al. 2021(1) | |  |  |
| Health Diary | Generic | Self-report | | English, French, German, Italian | Patient and caregiver | NA/NA | | | Work-time loss and absenteeism | NA | | Yes, for work-time loss and absenteeism | | Yes, for HCA | | Mamiya et al. 2025(2) | |  |  |
| Health Productivity Scale (HPS) | Generic | Self-report | | English | Patient | NA/NA | | | Presenteeism | 30 days | | No | | No | | Hubens et al. 2021(1) | |  |  |
| Health-Related Productivity Loss Instrument (HPLI) | Generic | Self-report | | English | Patient | NA/NA | | | Absenteeism and presenteeism | 2 weeks | | Yes, for absenteeism | | No | | Hubens et al. 2021(1) | |  |  |
| Job-Stress-Related Presenteeism Scale (JSRPS) | Generic | Self-report | | English | Patient | NA/NA | | | Presenteeism | NA | | No | | No | | Hubens et al. 2021(1) | |  |  |
| Lam Employment Absence and Productivity Scale (LEAPS) | Generic | Self-report | | English | Patient | NA/NA | | | Absenteeism and presenteeism | 2 weeks | | Yes, for absenteeism and presenteeism | | No | | Hubens et al. 2021(1) | |  |  |
| MS Questionnaire | Disease-specific | Interview | | Spanish (Panama) | Patient | NA/NA | | | Absenteeism | From symptom onset until today | | Yes, for absenteeism | | No | | Hubens et al. 2021(1) | |  |  |
| Nurses Work Functioning Questionnaire (NWFQ) | Generic | Self-report | | English | Patient | NA/NA | | | Presenteeism | 4 weeks | | No | | No | | Hubens et al. 2021(1) | |  |  |
| Otago Cost and Consequences Questionnaire (OCC-Q) | Disease-specific | Self-report | | English | Patient | NA/NA | | | Absenteeism and presenteeism | 1 month | | Yes, for absenteeism | | No | | Hubens et al. 2021(1) | |  |  |
| Patient Self-Assessment Questionnaire & Diary (SAQ) | Disease-specific | Self-report | | English | Patient | NA/NA | | | Absenteeism | NA | | Yes, for absenteeism | | No | | Hubens et al. 2021(1) | |  |  |
| Presenteeism Questions by Aronsson et al. | Generic | Self-report | | English | Patient | NA/NA | | | Presenteeism | 12 months | | No | | No | | Hubens et al. 2021(1) | |  |  |
| Productivity and Disease Questionnaire (PRODISQ) | Generic | Self-report | | Dutch | Patient | NA/NA | | | Absenteeism and presenteeism | 3 months | | Yes, for absenteeism | | Yes, for FCA | | Hubens et al. 2021(1) | |  |  |
| Proforma for Cost of illness for Irritable Bowel Syndrome | Disease-specific | Self-report | | English | Patient | NA/NA | | | Absenteeism | NA | | Yes, for absenteeism | | No | | Hubens et al. 2021(1) | |  |  |
| Question by Vänni et al. | Generic | Self-report | | English | Patient | NA/NA | | | Presenteeism | 2 years | | No | | No | | Hubens et al. 2021(1) | |  |  |
| Questionnaire by Agboola et al. | Disease-specific | Self-report | | English | Patient | NA/NA | | | Presenteeism, absenteeism, and unpaid work | NA | | Yes, for absenteeism | | No | | Hubens et al. 2021(1) | |  |  |
| Questionnaire by Tanvejsilp et al. | Disease-specific | Self-report | | English, Thai | Patient | NA/NA | | | Absenteeism | 1 day | | Yes, for absenteeism | | No | | Hubens et al. 2021(1) | |  |  |
| Resource Use Questionnaire (RUQ) | Generic | Self-report | | English | Patient | NA/NA | | | Absenteeism and presenteeism | NA | | Yes for absenteeism | | Yes, for HCA | | Mamiya et al. 2025(2) | |  |  |
| Resource Use Questionnaire-Preschool (RUQ-P) | Disease-specific | Interview | | English, French (Canada) | Parent | NA/NA | | | Absenteeism, presenteeism, and unpaid work | 12 months | | Yes, for absenteeism and unpaid work | | No | | Hubens et al. 2021(1) | |  |  |
| Resource Utilization in Dementia (RUD) | Disease-specific | Self-report | | Over 40 languages, including English | Caregiver | NA/NA | | | Absenteeism | 30 days | | Yes, for absenteeism | | No | | Hubens et al. 2021(1) | |  |  |
| Resource utilization questionnaire | Generic | Self-report | | Over 60 languages, including English | Patient | NA/NA | | | Absenteeism and presenteeism | 3 months | | Yes for absenteeism and presenteeism | | Yes, for HCA | | Mamiya et al. 2025(2) | |  |  |
| Scoliosis Research Society Outcomes Questionnaire (SRS-22r) | Generic | Self-report | | English, Chinese (traditional), Dutch, Italian, Norwegian, Spanish, Greek, Japanese, Korean, Persian, Thai, and Turkish | Patient | Yes/NA | | | Work-time loss | NA | | No | | No | | Mamiya et al. 2025(2) | |  |  |
| Single item presenteeism question (SIPQ) | Disease-specific | Self-report | | English, Persian | Patient | NA/NA | | | Presenteeism | 30 days | | Yes, for presenteeism | | No | | Hubens et al. 2021(1) | |  |  |
| Survey by Mumford et al. | Disease-specific | Self-report | | English | Parent | NA/NA | | | Absenteeism and unpaid work | Variable period from admission date | | Yes, for absenteeism and unpaid work | | No | | Hubens et al. 2021(1) | |  |  |
| The cost diary by Goossens et al. | Generic | Self-report | | English | Patient | NA/NA | | | Absenteeism and unpaid work | 1 week | | Yes, for absenteeism and unpaid work | | No | | Hubens et al. 2021(1) | |  |  |
| Utrecht Work Engagement Scale | Generic | Self-report | | English, Spanish, Portuguese, Finnish, Greek, Thai, Vietnamese, Sinhala, Chinese, Japanese, Serbian, Sinhala | Patient | NA/NA | | | Presenteeism | One month | | Yes for presenteeism | | No | | Zhang et al. 2023(3) | |  |  |
| WHO generic patient with tuberculosis cost survey instrument | Disease-specific | Interview | | English, Arabic, Indonesian, French, Swahili, Portuguese (Brazilian) | Patient | Yes/NA | | | Work-time loss | NA | | Yes. for work-time loss | | Yes, for HCA | | Mamiya et al. 2025(2) | |  |  |
| Work absence question by Wynne-Jones et al. | Disease-specific | Self-report | | English | Patient | NA/NA | | | Absenteeism | 4 months | | Yes, for absenteeism | | No | | Hubens et al. 2021(1) | |  |  |
| Work and Health Interview (WHI) | Generic | Interview | | English | Patient | NA/NA | | | Absenteeism and presenteeism | 2 weeks | | Yes, for absenteeism and presenteeism | | No | | Hubens et al. 2021(1) | |  |  |
| Work Productivity and Activity Impairment plus Classroom Impairment (WPAI-CIQ-SHP, version 2) | Disease-specific | Self-report | | More than 20 languages including English | Patient | NA/NA | | | Absenteeism, presenteeism, and unpaid work | 7 days | | Yes, for absenteeism, presenteeism, and unpaid work | | No | | Hubens et al. 2021(1) | |  |  |
| Work Productivity Short Inventory (WPSI) | Disease-specific | Self-report | | English | Patient | NA/NA | | | Absenteeism and presenteeism | 1 year/3 months/2 weeks | | Yes, for absenteeism and presenteeism | | No | | Hubens et al. 2021(1) | |  |  |
| Workplace Outcome Suite | Generic | Self-report | | English, Greek, Mandarin, Portuguese, Spanish | Patient | NA/NA | | | Absenteeism and presenteeism | 1 week | | Yes for absenteeism and presenteeism | | Yes, for HCA | | Zhang et al. 2023(3) | |  |  |

Abbreviations: FCA: friction cost approach; HCA: human capital approach; NA: not applicable.

**REFERENCES**

1.	**Hubens K, Krol M, Coast J & et al.** (2021) Measurement Instruments of Productivity Loss of Paid and Unpaid Work: A Systematic Review and Assessment of Suitability for Health Economic Evaluations From a Societal Perspective. *Value Health* **24**, 1686-99.

2.	**Mamiya H, Wattanakamolkul K, Li N, et al.** (2025) Including Productivity as an Element to Reflect Value of the Treatment: A Systematic Review of Published Health Economic Evaluations. *Pharmacoeconom Open* **24**, 24.

3.	**Zhang W, Tocher P, L’Heureux J, Sou J & Sun H** (2023) Measuring, analyzing, and presenting work productivity loss in randomized controlled trials: a scoping review. *Value in Health* **26**, 123-37.
